# Dihydroartemisinin inhibits the Raf/ERK/MEK and PI3K/AKT pathways in glioma cells

**DOI:** 10.3892/ol.2021.12636

**Published:** 2021-03-15

**Authors:** Wei Du, Changhe Pang, Yake Xue, Qingjun Zhang, Xinting Wei

Oncol Lett 10: 3266-3270, 2015; DOI: 10.3892/ol.2015.3699

Following the publication of the above article, an interested reader drew to the authors’ attention that lane 1 of the pMEK/BT325 cells panel in Fig. 3A appeared strikingly similar to lane 1 of the BCL-2 / C6 cells panel in Fig. 5B, albeit the protein band was inverted. Furthermore, lanes 1–3 of the MEK / C6 cells panel in Fig. 3B looked strikingly similar to lanes 1–3 of the GAPDH / BT325 cells panel in Fig. 5A.

The authors re-examined their original data, and realized that some of the data featured in Fig. 3 had been assembled incorrectly owing to the large number of proteins that had been investigated, and given the fact that not only one person was responsible for generating these data. The corrected version of Fig. 3 is shown opposite. Note that the corrections made to this figure do not affect the overall conclusions reported in the paper. The authors are grateful to the Editor of *Oncology Letters* for allowing them the opportunity to publish this corrigendum, and apologize to the readership for any inconvenience caused.

## Figures and Tables

**Figure 3.**
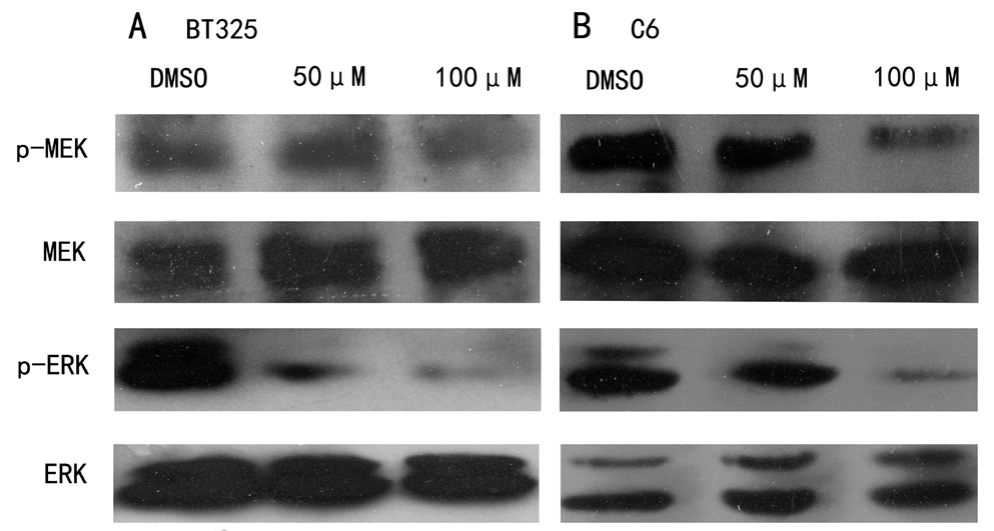
DHA inhibits RAF/MEK/ERK and PI3K/AKT signaling pathways in (A) BT325 and (B) C6 cells. Cells were treated with the indicated concentrations of DHA for 24 h. Western blot analysis was performed using specific antibodies against the indicated proteins.

